# ATP evokes Ca^2+^ signals in cultured foetal human cortical astrocytes entirely through G protein‐coupled P2Y receptors

**DOI:** 10.1111/jnc.14119

**Published:** 2017-08-02

**Authors:** Margit S. Muller, Colin W. Taylor

**Affiliations:** ^1^ Department of Pharmacology University of Cambridge Cambridge UK

**Keywords:** P2X receptor, P2Y_1_ receptor, P2Y_2_ receptor, phospholipase C, purinoceptor, store‐operated Ca^2+^ entry

## Abstract

Extracellular ATP plays important roles in coordinating the activities of astrocytes and neurons, and aberrant signalling is associated with neurodegenerative diseases. In rodents, ATP stimulates opening of Ca^2+^‐permeable channels formed by P2X receptor subunits in the plasma membrane. It is widely assumed, but not verified, that P2X receptors also evoke Ca^2+^ signals in human astrocytes. Here, we directly assess this hypothesis. We showed that cultured foetal cortical human astrocytes express mRNA for several P2X receptor subunits (P2X_4_, P2X_5_, P2X_6_) and G protein‐coupled P2Y receptors (P2Y_1_, P2Y_2_, P2Y_6_, P2Y_11_). In these astrocytes, ATP stimulated Ca^2+^ release from intracellular stores through IP_3_ receptors and store‐operated Ca^2+^ entry. These responses were entirely mediated by P2Y_1_ and P2Y_2_ receptors. Agonists of P2X receptors did not evoke Ca^2+^ signals, and nor did ATP when Ca^2+^ release from intracellular stores and store‐operated Ca^2+^ entry were inhibited. We conclude that ATP‐evoked Ca^2+^ signals in cultured human foetal astrocytes are entirely mediated by P2Y_1_ and P2Y_2_ receptors, with no contribution from P2X receptors.

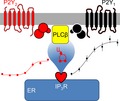

**Abbreviations used**
[Ca^2+^]_i_: intracellular free Ca^2+^ concentration
2‐APB: 2‐aminoethoxydiphenyl borate
ER: endoplasmic reticulum
GFAP: glial fibrillary acidic protein
HBS: HEPES‐buffered saline
IP_3_: inositol 1,4,5‐trisphosphate
pEC_50_: −log of the half‐maximally effective drug concentration
PLC: phospholipase C
SOCE: store‐operated Ca^2+^ entry

Astrocytes comprise a diverse population of glial cells that express glial fibrillary acidic protein, synthesise and store glycogen granules and are linked to each other by gap junctions (Haydon 2001; Verkhratsky and Butt 2013). Astrocytes are abundant throughout the brain and spinal cord, where their roles include directing migration of neurons during development; release of extracellular matrix molecules and growth factors; secretion of neurotransmitters, including ATP and glutamate; regulation of the neuronal environmental; providing neurons with nutrients; and inactivation and recycling of neurotransmitters (Haydon 2001).

Astrocytes respond to many neurotransmitters, but ATP and glutamate are the most prominent, and both can evoke Ca^2+^ signals that trigger further release of ATP or glutamate. This interplay allows reciprocal interactions between astrocytes and neurons, and it contributes, alongside diffusion of inositol 1,4,5‐trisphosphate (IP_3_) through gap junctions, to regenerative propagation of ATP‐evoked Ca^2+^ signals between astrocytes (Haydon 2001). ATP signalling in astrocytes thereby contributes to diverse physiological and pathophysiological processes, including gliotransmitter release, cytokine expression, nociception, regulation of synaptic strength, astrogliosis, ischaemia‐induced injury and Alzheimer's disease (Franke *et al*. 2001; Duan *et al*. 2003; Pascual *et al*. 2005; Lammer *et al*. 2006; Delekate *et al*. 2014).

Responses to ATP are mediated by two families of P2 receptors. P2X receptors are ligand‐gated cation channels, which are permeable to Na^+^, K^+^ and Ca^2+^ (Burnstock and Kennedy 2011). The seven P2X receptor subtypes (P2X_1‐7_) form homo‐ or hetero‐trimeric channels. ATP is the major endogenous agonist for all P2X receptors (Soto *et al*. 1996; Nicke *et al*. 1998). P2Y receptors are G protein‐coupled receptors. Five of the eight P2Y receptor subtypes (P2Y_1_, P2Y_2_, P2Y_4_, P2Y_6_ and P2Y_11_) stimulate phospholipase C (PLC) through G_q_, and the others inhibit adenylyl cyclase through G_i_ (P2Y_12_, P2Y_13_ and P2Y_14_) (Alexander *et al*. 2011). Endogenous ligands of P2Y receptors include ADP, ATP, UDP (uridine 5′‐diphosphate), UTP (uridine 5′‐triphosphate) and UDP‐glucose (Jacobson and Muller 2016).

Despite acceptance of the importance of ATP‐evoked Ca^2+^ signals in astrocytes, the evidence derives almost entirely from rodents, where mRNA for most P2 receptors has been detected, and both P2X and P2Y receptors have been implicated in Ca^2+^ signalling (Fumagalli *et al*. 2003; Verkhratsky *et al*. 2009). However, there is a widespread assumption that most ATP‐evoked Ca^2+^ signals in rodent astrocytes are mediated by P2X_1/5_ and P2X_7_ receptors (Lalo *et al*. 2008, 2011). The evidence implicating P2X_7_ receptors is controversial and derives largely from analyses of reactive astroctyes (Sim *et al*. 2004; Verkhratsky *et al*. 2009; Oliveira *et al*. 2011), where morphology and function are changed by the inflammatory mediators that are inevitably released during preparation of brain slices (Takano *et al*. 2014; Ben Haim *et al*. 2015). Furthermore, in humans, the P2X_5_ subunit is truncated and retained within the endoplasmic reticulum (ER) (Kotnis *et al*. 2010). In cultured rodent astrocytes, P2Y_1_ and P2Y_2_ receptors, and to a lesser extent P2Y_4_ receptors, can also initiate ATP‐evoked Ca^2+^ signals (Verkhratsky *et al*. 2009). Hence, even in rodent astrocytes, the identities of the receptors that mediate ATP‐evoked Ca^2+^ signals are unresolved (Fumagalli *et al*. 2003; Verkhratsky *et al*. 2009).

In human astrocytes, the receptors that mediate ATP‐evoked Ca^2+^ signals are unknown. There has been no complete or quantitative analysis of mRNA expression levels for P2 receptors, although in cultures of human astrocytes mRNAs for P2Y_1_, P2Y_2_, P2Y_4_, P2X_4_, P2X_5_ and P2X_7_ receptors were detected (John *et al*. 2001; Narcisse *et al*. 2005; Hashioka *et al*. 2014). The only P2 receptor protein shown to be expressed is the P2X_7_ subunit, but in healthy astrocytes it was exclusively expressed on intracellular membranes, and in brain sections it was detected only in diseased tissue (Narcisse *et al*. 2005). In the only analyses of Ca^2+^ signals, 2‐MeS‐ATP and UTP evoked Ca^2+^ signals in cultured human astrocytes, but the receptor pharmacology was not further defined (John *et al*. 1999). In another study, an agonist of P2X receptors (BzATP), which also stimulates P2Y_11_ receptors (Communi *et al*. 1999), evoked a convincing Ca^2+^ signal only in reactive astrocytes (Narcisse *et al*. 2005). Hence, the common but unverified, assumption that ATP‐evoked Ca^2+^ signals in healthy human astrocytes are largely mediated by P2X receptors requires further investigation (Burnstock 2008; Illes *et al*. 2012).

In this study, we define the receptors responsible for ATP‐evoked Ca^2+^ signals in human astrocytes. We used cultures of foetal cortical human astrocytes to quantify mRNA expression for all P2 receptors, and we identified the P2 receptors through which ATP evokes Ca^2+^ signals. There are limitations to the use of cultured cells, but for human brain tissue, it provides the only practicable means of directly measuring cytosolic Ca^2+^ signals. Furthermore, it avoids the persistent astrogliosis caused by the traumatic injury and hypoxia inherent in preparing brain slices, which has been shown to affect expression of P2 receptors (Narcisse *et al*. 2005; Takano *et al*. 2014). Our results show that cultured human foetal astrocytes express mRNA for several P2X and P2Y receptors, but the Ca^2+^ signals evoked by ATP are entirely mediated by P2Y_1_ and P2Y_2_ receptors.

## Materials and methods

### Materials

Fura‐2 AM was from Invitrogen (Paisley, UK). Fluo‐8 AM was from Stratech Scientific (Suffolk, UK). MRS2365 ((N)‐methanocarba‐2‐methylthio‐adenosine‐5′‐diphosphate), MRS2179 (2′‐deoxy‐*N* ^6^‐methyladenosine 3′,5′‐bisphosphate), U73122 (1‐[6‐[[(17β)‐3‐methoxyestra‐1,3,5(10)‐trien‐17‐yl]amino]hexyl]‐1H‐pyrrole‐2,5‐dione), U73343 (1‐[6‐[[(17β)‐3‐methoxyestra‐1,3,5(10)‐trien‐17‐yl]amino]hexyl]‐2,5‐pyrrolidinedione), SKF96365 (1‐[2‐(4‐methoxyphenyl)‐2‐[3‐(4‐methoxyphenyl)propoxy]ethyl‐1*H*‐imidazole hydrochloride) and BTP‐2 (N‐[4‐[3,5‐bis(trifluoromethyl)‐1H‐pyrazol‐1‐yl]phenyl]‐4‐methyl‐1,2,3‐thiadiazole‐5‐carboxamide) were from Tocris (Bristol, UK). Fibronectin was from Merck Millipore (Watford, UK). Bovine serum albumin was from Europa Bioproducts Ltd (Cambridge, UK). 2′‐amino‐UTP (2′‐amino‐2′‐deoxyuridine‐5′‐triphosphate) and 2′‐thio‐UTP (2′‐thio‐2′‐deoxyuridine‐5′‐triphosphate) were from Trilink Biotechnologies (San Diego, CA, USA). Thapsigargin was from Bio Techne (Abingdon, UK). qPCR primers and related reagents were from Qiagen (Crawley, West Sussex, UK). All other reagents, including 2‐aminoethoxydiphenyl borate (2‐APB), ADP, ATP, probenecid, UDP and UTP were from Sigma‐Aldrich (Gillingham, UK). The properties of the drugs used are summarized in Table S1.

### Cell culture

Human astrocytes isolated from foetal cortex were supplied as frozen cells that had not been passaged (catalogue number CC‐2565, Lonza, Slough, UK). The cells were confirmed, by Lonza, to be free of infection with HIV‐1 and hepatitis B and C, and we confirmed that they were free of mycoplasma. Astrocytes were grown at 37°C in humidified air containing 5% CO_2_, using astrocyte growth medium (Lonza) supplemented with 3% foetal bovine serum. Astrocyte growth medium includes human epidermal growth factor, insulin, ascorbic acid, gentamycin and l‐glutamine. Cells were passaged using trypsin, according to the supplier's instructions, when they reached 70–80% confluence. Cells were used for up to four passages after receipt, during which they maintained an astrocyte‐like morphology and expressed glial fibrillary acidic protein, assessed by qPCR and immunostaining.

### Quantitative PCR

For quantitative PCR (qPCR), confluent cultures of astrocytes in 24‐well plates were lysed (200 μL cell processing buffer/well), mRNA was then isolated from the lysate (4 μL) and cDNA was synthesized using Fastlane cell cDNA kit (Qiagen,Crawley, UK). The cDNA was diluted fivefold with RNAase‐free water. Incubations for qPCR included Rotor‐Gene SYBR™ Green PCR master mix (10 μL), cDNA (5 μL), Quantitect primer assay (2 μL, Table S2) and RNAase‐free water (3 μL). In negative controls, the primers were omitted during qPCR or the reverse‐transcriptase was omitted during cDNA synthesis. A Rotor‐Gene 6000 thermocycler (Qiagen) was used for qPCR with a denaturation step (95°C, 5 min), 40 amplification cycles (5 s at 95°C, 10 s at 60°C) and then a melting curve (70–95°C). Expression of mRNA relative to glyceraldehyde 3‐phosphate dehydrogenase (GAPDH) was calculated from: Relative expression $=\frac{E^{-C_{T}^{\mathrm{PURINOCEPTOR}}}}{E^{-C_{T}^{\mathrm{GAPDH}}}}$, where E is the amplification efficiency, calculated as 10^m^, where m is the average increase in fluorescence for four cycles after the cycle threshold C_T_ for the indicated PCR product. The effectiveness of all primer pairs was verified using BioBank generic pooled cDNA (Primerdesign Ltd, Chandler's Ford UK). All primers included in this study amplified a single product from the BioBank pooled cDNA (Figure S1). The melting temperatures of all products amplified from cDNA from astrocytes were identical to the respective BioBank controls. Results are reported as means from cDNA samples independently obtained from three different cell cultures.

### Measurements of [Ca^2+^]_i_ in populations of astrocytes

Confluent cultures of astrocytes grown in fibronectin‐coated 96‐well plates (Greiner Bio‐One, Stonehouse, UK) were incubated at 20°C with fluo‐8 AM (4 μM) in HEPES‐buffered saline (HBS) containing probenecid (2.5 mM) (Di Virgilio *et al*. 1990). HBS had the following composition: 135 mM NaCl, 5.9 mM KCl, 1.2 mM MgCl_2_, 1.5 mM CaCl_2_, 11.5 mM glucose and 11.6 mM HEPES, pH 7.3. After 60 min, the cells were washed with HBS, incubated for 90 min in HBS containing probenecid (2.5 mM) to allow de‐esterification of the indicator, washed and then used immediately for experiments. All experiments were performed at 20°C, to avoid extrusion and intracellular compartmentalization of fluo‐8, in HBS without probenecid. Where indicated, Ca^2+^‐free HBS containing BAPTA (1,2‐bis(o‐aminophenoxy)ethane‐N,N,N′,N′‐tetraacetic acid, final concentration 2.5 mM) was added immediately before stimulation to reduce the free [Ca^2+^] of HBS to < 100 nM.

Fluorescence (excitation at 490 nm, emission at 520 nm) was recorded at 1.44‐s intervals using a FlexStation III fluorescence plate‐reader (MDS Analytical Technologies, Wokingham, UK), which allows automated fluid additions during the recording (Tovey *et al*. 2006). Fluorescence (F) was calibrated to intracellular free Ca^2+^ concentration ([Ca^2+^]_i_) from: ${\left[{\text{Ca}}^{2+}\right]}_{i}=K_{d}\frac{F-F_{\min}}{F_{\max}-F}$, where K_D_ is the equilibrium dissociation constant of fluo‐8 for Ca^2+^ (389 nM), F_min_ and F_max_ are the minimal and maximal fluorescence values determined after addition of Triton X‐100 (0.2%) in Ca^2+^‐free HBS with BAPTA (10 mM, F_min_) or ionomycin (10 μM) in normal HBS (F_max_).

### Measurement of [Ca^2+^]_i_ in single astrocytes

Almost confluent cultures of astrocytes grown on fibronectin‐coated eight‐well imaging slides (Thistle Scientific Ltd, Glasgow, UK) were loaded with fura‐2 by incubation with fura‐2 AM (2 μM) in HBS containing 2.5 mM probenecid (45 min, 20°C). After a further 45 min in the same medium without fura‐2 AM, the cells were used for experiments at 20°C in HBS without probenecid. Imaging was performed using an Olympus IX71 inverted fluorescence microscope with alternating excitation (340 nm and 380 nm) provided by a Xe‐arc lamp at 1‐s intervals. Emission was recorded at 510 nm using a Luca EMCCD camera (Andor Technology, Belfast, UK) and MetaFluor software (Molecular Devices, Sunnyvale, CA, USA). Background‐corrected ratios of F_340_/F_380_ fluorescence were used to determine whether ligands evoked increases in [Ca^2+^]_i_.

### Statistical analysis

For each experiment, the concentration‐response relationship was fitted to a logistic equation (GraphPad Prism 5, La Jolla, CA, USA) from which the maximal amplitude and pEC_50_ values (−log of the half‐maximally effective drug concentration) were determined. All analyses, except when otherwise stated, show pooled results from cells from two donors (Lonza lot numbers: 0000289765 and 0000402839). Key results from these and a third donor (0000514417) are shown individually in Figure S2. Results are presented as means ± SEM of values from at least three independent experiments. Sample sizes (n) refer to independent experiments.

## Results

### ATP evokes Ca^2+^ release and Ca^2+^ entry in cultured human foetal astrocytes

**Figure 1:**
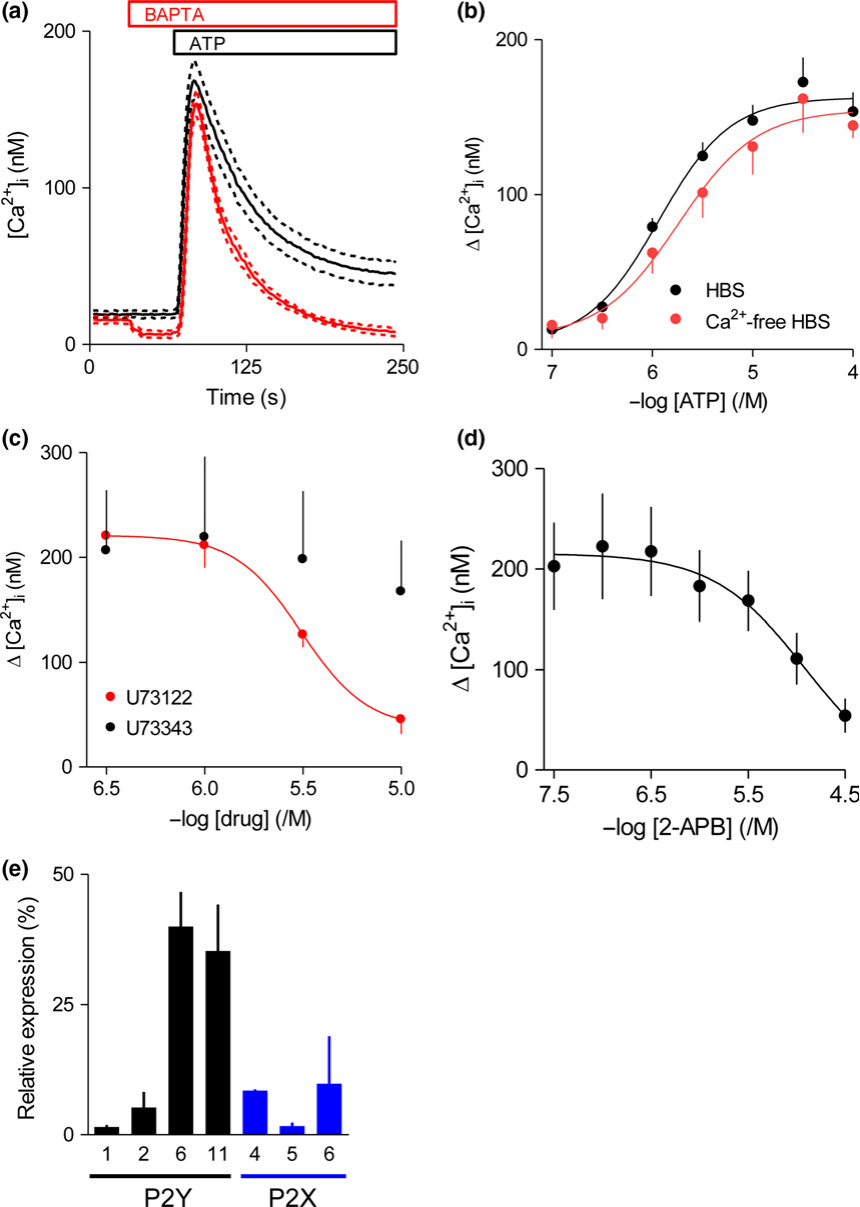
ATP stimulates Ca^2+^ release from intracellular stores and Ca^2+^ entry in cultured human foetal astrocytes. (a) Populations of fluo‐8‐loaded astrocytes were stimulated with ATP (100 μM) in HEPES‐buffered saline (HBS) (black; *n* = 8) or Ca^2+^‐free HBS (red; *n* = 5, the addition of BAPTA, final concentration 2.5 mM, to chelate extracellular Ca^2+^ is shown). Results show [Ca^2+^]_i_ as means (solid lines) ± SEM (dashed lines). (b) Summary results (means ± SEM) show effects of the indicated concentrations of ATP on the peak increase in [Ca^2+^]_i_ (Δ[Ca^2+^]_i_) in the presence (*n* = 8) or absence (*n* = 5) of extracellular Ca^2+^. (c) Effects of pre‐treatment (5 min) with the indicated concentrations of U73122 or U73343 in HBS on Δ[Ca^2+^]_i_ evoked by ATP (100 μM). (d) Similar analysis of the effects of pre‐treatment (5 min) with 2‐2‐aminoethoxydiphenyl borate (APB) in HBS. Results (c and d) show means ± SEM,* n* = 3. For clarity, only a single error bar is shown in (b) and (c). (e) Expression of mRNA for P2 receptors was measured by qPCR relative to mRNA for glyceraldehyde 3‐phosphate dehydrogenase (GAPDH). Results (means ± SEM from three independent samples, each measured in duplicate) are expressed as percentages of all P2 receptor mRNA. There was no detectable expression of mRNA for the remaining P2Y (4, 12–14) or P2X (1‐3, 7) receptor subtypes, although the primers used were all shown to be effective (Figure S1).

ATP evoked a concentration‐dependent increase in [Ca^2+^]_i_ (pEC_50_ = 5.94 ± 0.03, *n* = 8) in populations of cultured foetal human cortical astrocytes. Similar results and with similar sensitivities to ATP, but with Ca^2+^ signals of different amplitude, were observed in cells from three different donors (Figure S2A). Removal of extracellular Ca^2+^ affected neither the peak amplitude of the increase in [Ca^2+^]_i_ nor its sensitivity to ATP (pEC_50_ = 5.64 ± 0.08, *n* = 5), but the sustained phase of the response was abolished (Fig. 1a and b). These results establish that release of Ca^2+^ from intracellular stores and Ca^2+^ entry across the plasma membrane contribute to the ATP‐evoked Ca^2+^ signals. U73122, an inhibitor of phospholipase C (Bleasdale *et al*. 1990), but not its inactive analogue (U73343), caused a concentration‐dependent inhibition of the ATP‐evoked Ca^2+^ signals (Fig. 1c). 2‐APB, an antagonist at IP_3_Rs (Saleem *et al*. 2014), also inhibited ATP‐evoked Ca^2+^ signals (Fig. 1d). Neither 2‐APB nor U73122 completely blocked the response to ATP, but the range of useable concentrations is limited by off‐target effects of the inhibitors (Grierson and Meldolesi 1995; Mogami *et al*. 1997; Peppiatt *et al*. 2003). These results demonstrate that ATP‐evoked Ca^2+^ signals are at least substantially dependent on stimulation of PLC and IP_3_‐evoked release of Ca^2+^ from intracellular stores.

We used qPCR with primers demonstrated to selectively amplify mRNA encoding each of the human P2Y and P2X receptors (Table S2) to quantify expression of these mRNAs in cultured foetal human cortical astrocytes. The results confirmed expression of mRNA for four of the eight subtypes of P2Y receptors (P2Y_1_, P2Y_2_, P2Y_6_ and P2Y_11_) and three of the seven subunits of P2X receptors (P2X_4_, P2X_5_ and P2X_6_) (Fig. 1e). There was no detectable expression of mRNA for the remaining P2Y or P2X receptors, despite the proven effectiveness of the primers used (Figure S1).

### P2X receptors do not evoke Ca^2+^ signals

Since some P2Y receptors, but no P2X receptors, can stimulate PLC (Burnstock and Kennedy 2011), our results so far suggest a major (and perhaps exclusive) role for P2Y receptors in initiating ATP‐evoked Ca^2+^ signals in human astrocytes. This contrasts with the prominent role ascribed to P2X receptors in rodent astrocytes. We therefore assessed whether P2X receptors contribute to the Ca^2+^ signals evoked by ATP in human astrocytes.

**Figure 2:**
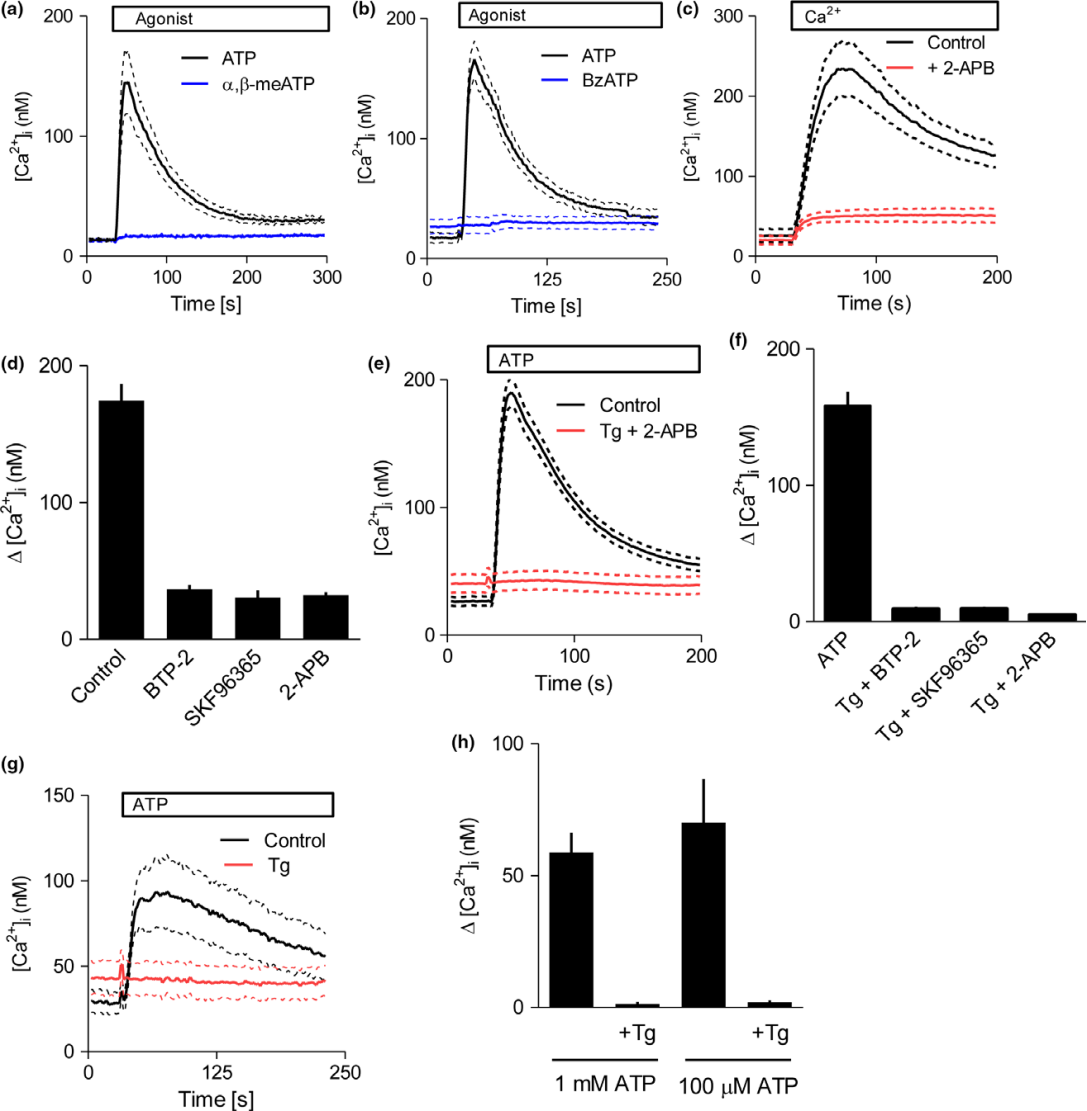
P2X receptors do not contribute to ATP‐evoked Ca^2+^ signals. (a and b) Populations of fluo‐8‐loaded astrocytes in HEPES‐buffered saline (HBS) were stimulated with ATP (100 μM, black), α,β‐meATP (30 μM, a; blue) or BzATP (100 μM, b; blue) as indicated. Results show [Ca^2+^]_i_ as means (solid lines) ± SEM (dashed lines; *n* = 3). (c) Cells were incubated with thapsigargin (5 μM, 15 min) in Ca^2+^‐free HBS alone or with 2‐2‐aminoethoxydiphenyl borate (APB) (100 μM). Traces (in the same format as a, *n* ≥ 4) show [Ca^2+^]_i_ after restoration of extracellular Ca^2+^ (2 mM). (d) Summary results (means ± SEM,* n* ≥ 4) show Δ[Ca^2+^]_i_ evoked by restoration of extracellular Ca^2+^ to thapsigargin‐treated cells after pre‐treatment (15 min) with 2‐APB (100 μM), SKF96365 (10 μM) or BTP‐2 (10 μM). (e) Astrocytes in HBS were stimulated with ATP (100 μM) alone or after pre‐treatment with thapsigargin (5 μM, 15 min) to deplete intracellular Ca^2+^ stores and 2‐APB (100 μM, 15 min) to inhibit store‐operated Ca^2+^ entry (SOCE). Traces are in the same format as a; *n* ≥ 6. (f) Summary results (means ± SEM,* n* ≥ 4) show Δ[Ca^2+^]_i_ evoked by ATP alone or after pre‐treatment with thapsigargin and the inhibitors shown (same concentrations as in d). (g) Ca^2+^ signals evoked by ATP in HBS (100 μM) alone or after pre‐treatment with thapsigargin (Tg, 5 μM, 15 min). Traces are in the same format as a; *n* = 3. (h) Summary results (means ± SEM,* n* = 3) show Δ[Ca^2+^]_i_ evoked by ATP alone (100 or 1 mM) or after pre‐treatment with thapsigargin.

We detected mRNA for P2X_4_, P2X_5_ and P2X_6_ receptor subunits in human astrocytes (Fig. 1e). P2X_4_ and P2X_5_, but not P2X_6_, subunits can form functional homo‐trimers (Torres *et al*. 1999). However, P2X_6_ subunits can form hetero‐trimers with P2X_4_ or P2X_5_ subunits, and the P2X_4/6_ structure has been shown to be functional (Le *et al*. 1998; Torres *et al*. 1999). Since our cultured astrocytes express mRNA for only three P2X receptor subunits, agonists that might otherwise inadequately distinguish between P2 receptors could be used to activate the candidate receptors (Table S1). Hence, astrocytes were stimulated in HBS with either α,β‐meATP, an agonist of human P2X_4_ and heteromeric P2X_4/6_ receptors (Le *et al*. 1998; Jones *et al*. 2000), or BzATP, an agonist of human P2X_5_ receptors (Bo *et al*. 2003). Neither α,β‐meATP nor BzATP, at concentrations more than sufficient to stimulate these P2X receptors (Le *et al*. 1998; Jones *et al*. 2000; Bo *et al*. 2003), evoked an increase in [Ca^2+^]_i_ (Fig. 2a and b). In these experiments, the peak increases in [Ca^2+^]_i_ (Δ[Ca^2+^]_i_) evoked by α,β‐meATP and BzATP were 5 ± 0 nM and 5 ± 3 nM respectively (*n* = 3); the parallel measurements of ATP‐evoked Δ[Ca^2+^]_i_ were 132 ± 26 nM and 150 ± 16 nM. We avoided higher concentrations of α,β‐meATP (100 μM) and BzATP (300 μM) because they evoked Ca^2+^ signals in Ca^2+^‐free HBS (not shown).

We next attempted to eliminate the Ca^2+^ signals evoked by P2Y receptors to unmask any possible contribution from P2X receptors. This required inhibition of both the Ca^2+^ release and Ca^2+^ entry components of the response evoked by P2Y receptors (Fig. 1a and b). Thapsigargin, which inhibits Ca^2+^ pumps in the ER, is commonly used to deplete the ER of Ca^2+^ and to thereby stimulate store‐operated Ca^2+^ entry (SOCE) (Parekh and Putney 2005). We confirmed that thapsigargin stimulated SOCE in human astrocytes (Fig. 2c). Pre‐treatment of astrocytes with three structurally unrelated inhibitors of SOCE, BTP‐2 (10 μM), SKF96365 (10 μM) and 2‐APB (100 μM) (Bootman *et al*. 2002; Liou *et al*. 2005; Ohga *et al*. 2008) almost abolished the SOCE evoked by thapsigargin (Fig. 2d). Although 2‐APB inhibits both IP_3_R and SOCE, its effects on thapsigargin‐evoked Ca^2+^ entry are probably due to it inhibiting formation of the STIM1 puncta that stimulate SOCE (DeHaven *et al*. 2008).

In astrocytes pre‐treated with thapsigargin to deplete intracellular Ca^2+^ stores and so prevent IP_3_‐evoked Ca^2+^ release, and with BTP‐2, SKF96365 or 2‐APB present to inhibit SOCE, a normally maximally effective concentration of ATP (100 μM) had no significant effect on [Ca^2+^]_i_ (Fig. 2e and f). Similar results were observed in cells from all three donors (Figure S2B). These results confirm that the Ca^2+^ entry evoked by ATP is likely mediated by SOCE, and that there is no additional response to ATP mediated by P2X receptors.

To exclude any possible off‐target effects of the SOCE inhibitors on P2X receptors, we compared the effects of ATP in HBS on astrocytes with and without prior thapsigargin treatment. This experiment is practicable because the amplitude of the Ca^2+^ signal evoked by SOCE decays relatively quickly in the continued presence of extracellular Ca^2+^ (Fig. 2c), such that the small residual SOCE‐mediated Ca^2+^ signal detected after 15 min would not obscure a response to ATP. Under these conditions, addition of ATP (100 μM or 1 mM) to thapsigargin‐treated cells in normal HBS had no significant effect on [Ca^2+^]_i_ (Fig. 2g and h). The lack of response to such high concentrations of ATP excludes a role for P2X receptors, including P2X_7_ receptors which have low affinity for ATP (Surprenant *et al*. 1996). These results demonstrate that P2X receptors make no detectable contribution to the Ca^2+^ signals evoked by ATP in cultured human cortical astrocytes, despite evidence that the cells express mRNA for three P2X receptor subunits (Fig. 1e).

An increase in [Ca^2+^]_i_ has been reported to stimulate translocation of P2X_4_ receptors from intracellular membranes to the plasma membrane (Qureshi *et al*. 2007; Vacca *et al*. 2009). We therefore considered whether release of Ca^2+^ from intracellular stores might stimulate a similar translocation of P2X receptors in human astrocytes and thereby allow ATP to sequentially activate P2Y and then P2X receptors. However, when astrocytes were first stimulated with ADP to activate P2Y (but not P2X) receptors, there was the expected increase in [Ca^2+^]_i_, but subsequent addition of α,β‐meATP to stimulate P2X receptors (30 μM after 5 min) evoked no further increase in [Ca^2+^]_i_ (Figure S3).

Collectively, these results demonstrate that the Ca^2+^ signals evoked by ATP in cultured human cortical astrocytes are entirely mediated by P2Y receptors with no detectable contribution from P2X receptors.

### P2Y_1_ and P2Y_2_ receptors mediate ATP‐evoked Ca^2+^ signals

All four of the P2Y receptor subtypes for which mRNA was detected in human astrocytes (P2Y_1_, P2Y_2_, P2Y_6_ and P2Y_11_) are coupled to G_q/11_ and can thereby stimulate PLC. We used ligands that distinguish between the subtypes for which mRNA was detected to resolve the contributions of different P2Y receptors to the ATP‐evoked Ca^2+^ signals (Table S1).

**Figure 3:**
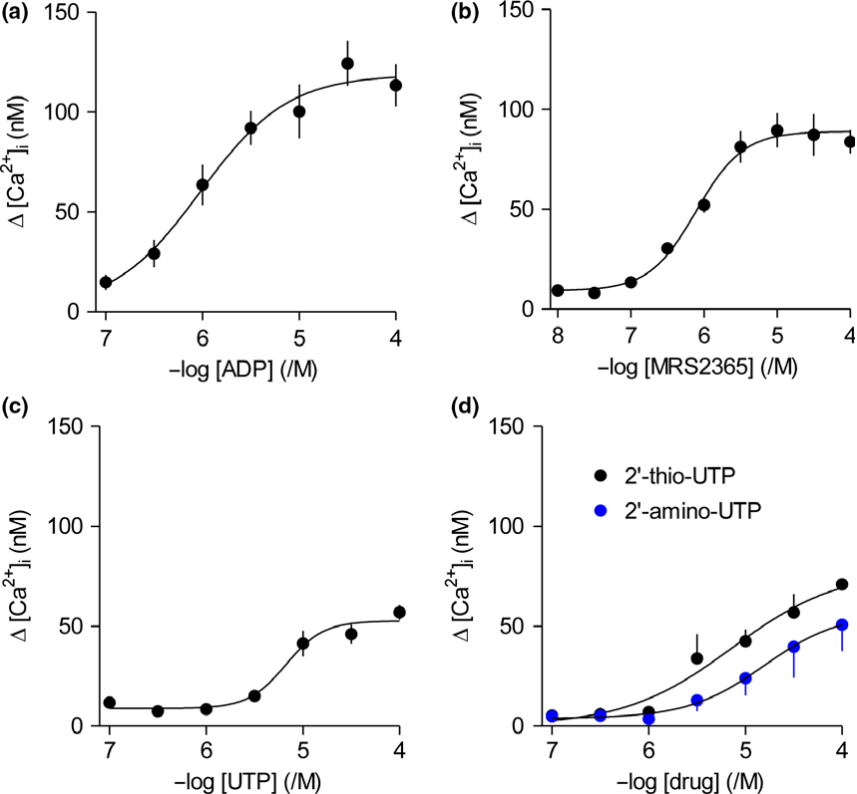
ATP‐evoked Ca^2+^ signals are mediated by P2Y_1_ and P2Y_2_ receptors. (a–d) Concentration‐dependent effects of the indicated agonists on Δ[Ca^2+^]_i_ were determined in normal HEPES‐buffered saline (HBS): ADP (P2Y_1_ and P2Y_11_) (a, *n* = 5), MRS2365 (P2Y_1_) (b, *n* = 5), UTP (P2Y_2_ and P2Y_6_) (c, *n* = 5), 2’‐thio‐UTP and 2’‐amino‐UTP (P2Y_2_) (d, *n* = 3).

ADP is an agonist of P2Y_1_, but not of P2Y_2_ or P2Y_11_ receptors. ADP caused a concentration‐dependent increase in [Ca^2+^]_i_ (pEC_50_ = 6.00 ± 0.11, *n* = 3) (Fig. 3a). Since ADP might also activate P2Y_6_ receptors (Communi *et al*. 1996), we also used MRS2365, a selective agonist of P2Y_1_ receptors (Table S1). MRS2365 evoked a concentration‐dependent increase in [Ca^2+^]_i_ (pEC_50_ = 6.20 ± 0.19, *n* = 5) and the maximal amplitude of the response was similar to that evoked by ADP (Fig. 3b). UDP is a potent agonist of P2Y_6_ receptors, but not of P2Y_1_, P2Y_2_ or P2Y_4_ receptors (Table S1). UDP had no effect on [Ca^2+^]_i_ (Figure S4A). Hence, P2Y_1_ receptors, but not P2Y_6_ receptors, contribute to the Ca^2+^ signals evoked by ATP.

UTP is a potent agonist of P2Y_2_ and P2Y_11_ receptors, but not of P2Y_1_ receptors (White *et al*. 2003). UTP caused a concentration‐dependent increase in [Ca^2+^]_i_ (pEC_50_ = 4.86 ± 0.18, *n* = 5) (Fig. 3c). The effect of UTP on [Ca^2+^]_i_ was mimicked by two additional agonists of P2Y_2_ receptors: 2′‐amino‐UTP (pEC_50_ = 4.57 ± 0.22, *n* = 3) and 2′‐thio‐UTP (pEC_50_ = 4.87 ± 0.47, *n* = 3) (Fig. 3d), but not by the related analogue, 2′‐azido UTP, which is a selective agonist of P2Y_4_ receptors (Jacobson *et al*. 2006) (Figure S5). NF546, a selective agonist of P2Y_11_ receptors, had no effect on [Ca^2+^]_i_ (Figure S4B). Hence, P2Y_2_ receptors also contribute to the ATP‐evoked Ca^2+^ signals. MRS2179 is a selective antagonist of P2Y_1_ receptors, and as expected, it caused a rightward shift of the concentration‐response relationship for the Ca^2+^ signals evoked by MRS2365, a selective agonist of P2Y_1_ receptors. In the presence of MRS2179 (5 μM), the ΔpEC_50_ value for MRS2365 was 0.64 ± 0.26 (where ΔpEC_50_ = $p_{{\mathrm{EC}}_{50}}^{+\mathrm{MRS}2179}-p_{{\mathrm{EC}}_{50}}^{\mathrm{control}}$; mean ± SEM, *n* = 6) (Figure S6A). In keeping with the ability of ATP to evoke Ca^2+^ signals through both P2Y_1_ and P2Y_2_ receptors, MRS2179 (5 μM) caused a smaller shift in the ΔpEC_50_ value for the Ca^2+^ signals evoked by ATP (0.47 ± 011, *n* = 7) (Figure S6B). The shifts in pEC_50_ values caused by MRS2179 were statistically significant (unpaired Student's *t*‐test, *p *=* *0.003 and 0.048 for ATP and MRS2365 respectively). We conclude that P2Y_1_ and P2Y_2_ receptors, but not P2Y_6_ or P2Y_11_ receptors, evoke Ca^2+^ signals in cultured foetal human astrocytes.

To determine whether activation of P2Y_1_ receptors (with ADP) and of P2Y_2_ receptors (with UTP) are entirely responsible for the Ca^2+^ signals evoked by ATP, we compared the maximal amplitudes of the responses evoked by the three stimuli in parallel measurements. The Δ[Ca^2+^]_i_ evoked by ATP, ADP and UTP were 142 ± 5 nM, 88 ± 11 nM and 51 ± 13 nM (*n* = 3) respectively. Hence, the sum of the responses to ADP and UTP (139 ± 25 nM) was not significantly different from the response evoked by ATP (142 ± 5 nM). These results confirm that ATP evokes Ca^2+^ signals through P2Y_1_ and P2Y_2_ receptors, but they do not resolve whether the two receptors are expressed in different cells or whether both contribute to the responses in individual cells. We therefore examined the responses of single fura‐2‐loaded cells to ATP, UTP and ADP.

In these single‐cell analyses, 87 ± 6% of cells responded to ATP (101 cells, 4 independent fields), 65 ± 5% responded to ADP (502 cells, 13 fields) and 41 ± 6% responded to UTP (386 cells, 11 fields), suggesting that at least 22% of ATP‐responsive cells express both P2Y_1_ and P2Y_2_ receptors. Analyses of responses to sequential stimulation with ADP and UTP revealed that 59 ± 8% of the cells that responded to ADP then responded to UTP (204 cells, 7 fields), while 92 ± 6% of cells that responded to UTP responded to a subsequent challenge with ADP (152 cells, 5 fields), suggesting that about half of the cells responded to both stimuli. These results demonstrate that most cells respond to ATP and that many express both P2Y_1_ and P2Y_2_ receptors. We considered whether autocrine release of ATP might contribute to the sustained phase of the Ca^2+^ signal evoked by selective activation of P2Y receptors. This seems unlikely, since in cell populations the relative amplitudes of the initial and sustained phases were similar for cells stimulated with ATP to activate all P2Y receptors or with ADP to activate only P2Y_1_ receptors (Fig. 1a and S3). Furthermore, in our single‐cell analyses, none of the cells that failed to respond initially to selective activation of P2Y_1_ receptors (176 cells) or P2Y_2_ receptors (228 cells) responded during the next 3–6 min with a detectable increase in [Ca^2+^]_i_.

## Discussion

We provide the first complete quantitative analysis of mRNA expression for P2 receptors in cultured foetal human cortical astrocytes, and a comprehensive pharmacological characterization of ATP‐evoked Ca^2+^ signals. We showed that mRNAs for four P2Y receptors (P2Y_6_ ~ P2Y_11_ > P2Y_2_ > P2Y_1_) and three P2X receptor subunits (P2X_6_ > P2X_4_ > P2X_5_) are expressed. There was no detectable mRNA for any of the remaining P2 receptors (Fig. 1e). The expression pattern is broadly consistent with previous studies of cultured human astrocytes from both adult (Hashioka *et al*. 2014) and foetal tissue (John *et al*. 2001; Narcisse *et al*. 2005), which examined mRNA for only seven of the fifteen P2 receptors, and detected mRNA for P2Y_1_, P2Y_2_, P2Y_4_, P2X_4_, P2X_5_ and P2X_7_ receptors. The notable differences are the absence of mRNA for P2Y_4_ and P2X_7_ receptors in our analyses, with the latter perhaps explained by the presence of fewer reactive astrocytes in our analysis (Narcisse *et al*. 2005). Neither we nor others have verified the relationship between mRNA and protein expression in human astrocytes because the P2 receptor‐selective antibodies generally lack specificity (Sim *et al*. 2004; Takano *et al*. 2014).

In keeping with many analyses of rodent astrocytes, ATP evoked an increase in [Ca^2+^]_i_ in both confluent populations of human cultured foetal astrocytes and sub‐confluent single cells (Verkhratsky *et al*. 2009). In human astrocytes, the initial response to ATP was because of Ca^2+^ release from intracellular stores through IP_3_ receptors (Fig. 1a–d), but the sustained response required Ca^2+^ entry across the plasma membrane. The Ca^2+^ entry had pharmacological properties typical of SOCE (Fig. 2e and f). In most cells, receptors that stimulate PLC usually activate SOCE (Parekh and Putney 2005), and in rodent astrocytes P2Y receptors have been shown to evoke Ca^2+^ entry by stimulating PLC (Fumagalli *et al*. 2003), but SOCE evoked by P2Y receptors has not, to the best of our knowledge, been previously reported for human astrocytes. These results are not consistent with the prominent role ascribed to P2X receptors in rodent astrocytes. Since mRNAs for three P2X receptor subunits were expressed in human astrocytes, we looked more closely to determine whether there was any underlying contribution from P2X receptors to ATP‐evoked Ca^2+^ signals. ATP analogues that would be expected to stimulate human P2X receptors assembled from P2X_4_, P2X_5_ or P2X_6_ subunits (α,β‐meATP and BzATP) did not increase [Ca^2+^]_i_ (Fig. 2a and b). Furthermore, under conditions where responses from IP_3_ receptors and SOCE were inhibited, there was no response to ATP (Fig. 2e and f). We confirmed that this lack of effect of ATP was not due to off‐target effects of the inhibitors used to block SOCE (Fig. 2g and h). Hence, whether assessed using ATP analogues selective for P2X receptors or ATP itself, there is no evidence that P2X receptors evoke Ca^2+^ signals in cultured human foetal astrocytes. Finally, we considered whether the IP_3_‐evoked Ca^2+^ signal might stimulate translocation of intracellular P2X_4_ receptors to the plasma membrane (Qureshi *et al*. 2007; Vacca *et al*. 2009), but we found no evidence to suggest that Ca^2+^ release and SOCE unmasked a response to P2X receptors (Figure S3).

The only published argument suggesting a role for P2X receptors in Ca^2+^ signalling in normal human astrocytes derives from their expression of mRNA for some P2X receptor subunits (John *et al*. 2001; Narcisse *et al*. 2005; Hashioka *et al*. 2014). Our results demonstrate that although cultured foetal cortical human astrocytes express mRNA for some P2X receptor subunits (Fig. 1e), P2X receptors do not contribute to the Ca^2+^ signals evoked by ATP (Fig. 2). Instead, we have shown that two of the four P2Y receptor subtypes for which mRNA was detected, P2Y_1_ and P2Y_2_ receptors, are entirely responsible for ATP‐evoked Ca^2+^ signals (Fig. 3 and Figure S2). Our conclusion is consistent with a previous report in which two non‐selective analogues, UTP and 2‐MeS‐ATP, which would together activate P2Y_1_ and P2Y_2_ receptors, evoked Ca^2+^ signals in human astrocytes (John *et al*. 1999).

Our analyses of mRNA for P2X receptors were not predictive for expression of functional plasma membrane receptors. Others have also noted expression of mRNA for P2 receptors for which there was no corresponding functional response (Fumagalli *et al*. 2003). For P2X_5_ subunits, a likely explanation is that the human protein is truncated and retained in the ER, where it may also trap other P2X subunits with which it can oligomerize (P2X_4_ and P2X_6_) (Torres *et al*. 1999; Kotnis *et al*. 2010). For P2Y receptors too, the most abundant mRNAs (for P2Y_6_ and P2Y_11_) were not associated with expression of functional P2Y receptors. In rodents too, there is no functional response to P2Y_6_ receptors, although their mRNA is expressed (Fumagalli *et al*. 2003). These observations are relevant because mRNA expression in astrocytes has often been used to infer the likely identity of the receptors that mediate ATP‐evoked Ca^2+^ signals (Verkhratsky *et al*. 2009).

We conclude that in cultured foetal cortical human astrocytes, ATP evokes Ca^2+^ signals that are entirely mediated by P2Y_1_ and P2Y_2_ receptors, each of which stimulates PLC and thereby IP_3_‐evoked Ca^2+^ release and SOCE. Many astrocytes express both of these receptors, but some express only one or the other. We have not further explored this heterogeneity. Although mRNA for P2X receptor subunits is expressed, P2X receptors do not contribute to ATP‐evoked Ca^2+^ signals.

## Supporting information


**Figure S1.** Melting curves for qPCR analyses of the expression of purinoreceptor subtypes.
**Figure S2.** ATP evokes Ca^2+^ signals through P2Y receptors in astrocytes from three donors.
**Figure S3.** Stimulation of P2Y receptors does not cause translocation of functional P2X receptors to the plasma membrane.
**Figure S4.** Neither P2Y_6_ nor P2Y_11_ receptors evoke Ca^2+^ signals in cultured human foetal astrocytes.
**Figure S5.** 2′‐azido‐UTP does not evoke Ca^2+^ signals.
**Figure S6.** Effects of MRS2179, a selective antagonist of P2Y_1_ receptors, on the Ca^2+^ signals evoked by ATP and MRS2365.
**Table S1.** Properties of the drugs used.
**Table S2.** Primers used for qPCR analyses.
**Table S3.** Ca^2+^ signals evoked by P2Y‐selective agonists in cultured human foetal astrocytes.
