# Supplementary material for: ATP evokes Ca2+ signals in cultured foetal human cortical astrocytes entirely through G protein‐coupled P2Y receptors
Source: J Neurochem. 2017 Aug 2;142(6):876–85. doi: 10.1111/jnc.14119 (PMC5601250; doi:10.1111/jnc.14119)
Supplement: Supplementary file 1 — Figure S1. Melting curves for qPCR analyses of the expression of purinoreceptor subtypes. Figure S2. ATP evokes Ca2+ signals through P2Y receptors in astrocytes from three donors. Figure S3. Stimulation of P2Y receptors does not cause translocation of functional P2X receptors to the plasma membrane. Figure S4. Neither P2Y6 nor P2Y11 receptors evoke Ca2+ signals in cultured human foetal astrocytes. Figure S5. 2′‐azido‐UTP does not evoke Ca2+ signals. Figure S6. Effects of MRS2179, a selective antagonist of P2Y1 receptors, on the Ca2+ signals evoked by ATP and MRS2365. Table S1. Properties of the drugs used. Table S2. Primers used for qPCR analyses. Table S3. Ca2+ signals evoked by P2Y‐selective agonists in cultured human foetal astrocytes. [file JNC-142-876-s001.pdf]

SUPPORTING INFORMATION

ATP evokes Ca^{2+} signals in cultured foetal human cortical astrocytes entirely through G protein-coupled P2Y receptors

Margit S. Muller and Colin W. Taylor

Department of Pharmacology, University of Cambridge, Tennis Court Road, Cambridge, CB2 1PD, United Kingdom.

Table S1. Properties of the drugs used

**Drug**	**Action**[1]	**Target in context of detected mRNA**[2]
ADP	Agonist of $P2Y_1$, $P2Y_{12}$, $P2Y_{13}$, and possibly $P2Y_6$ receptors	Agonist of $P2Y_1$ and $P2Y_6$ receptors
ATP	Agonist of P2Y and P2X receptors	Agonist of P2Y and P2X receptors
2'-amino-UTP	Agonist of $P2Y_2$ receptors	Agonist of $P2Y_2$ receptors
2-APB	Inhibits IP_3R and SOCE	
BzATP	Agonist of all P2X subtypes, high affinity for $P2X_1$, most commonly used as a $P2X_7$ agonist, full agonist at human $P2X_5$ with affinity similar to ATP	Agonist of $P2X_5$ receptors
BTP-2	Inhibits SOCE	
2'-thio-UTP	Agonist of $P2Y_2$ receptors	Agonist of $P2Y_2$ receptors
α,β-meATP	Agonist of $P2X_1$, $P2X_3$, human $P2X_4$ and $P2X_{4/6}$ receptors	Agonist of $P2X_4$ and $P2X_{4/6}$ receptors
MRS2365	Agonist of $P2Y_1$ receptors	Agonist of $P2Y_1$ receptors
NF546	Agonist of $P2Y_{11}$ receptors	Agonist of $P2Y_{11}$ receptors
SKF96365	Inhibits SOCE	
Thapsigargin	Inhibits ER/SR Ca^{2+}-ATPase (ER Ca^{2+} pump)	
U73122	Inhibits PLC	
U73343	Inactive analogue of U73122	
UDP	Agonist of $P2Y_6$ and $P2Y_{14}$ receptors	Agonist of $P2Y_6$ receptors
UTP	Agonist of $P2Y_2$, $P2Y_4$ and $P2Y_{11}$ receptors	Agonist of $P2Y_2$ and $P2Y_{11}$ receptors

[1]The pharmacology of P2 receptor agonists is described in von Kugelgen & Hoffmann (2015), and Syed & Kennedy (2012).
[2]Targets of the agonists are described in the context of the P2X and P2Y receptors for which mRNA was detected in cultured foetal cortical human astrocytes (Fig. 1E).

Table S2. Primers used for qPCR analyses

Purinoceptor	Quantitect Primers
$P2Y_1$	Hs_PURINOCEPTORY1_1_SG
$P2Y_2$	Hs_PURINOCEPTORY2_1_SG
$P2Y_4$	Hs_PURINOCEPTORY4_1_SG
$P2Y_6$	Hs_PURINOCEPTORY6_2_SG
$P2Y_{11}$	Hs_PURINOCEPTORY11_1_SG
$P2Y_{12}$	Hs_PURINOCEPTORY12_2_SG
$P2Y_{13}$	Hs_PURINOCEPTORY13_1_SG
$P2Y_{14}$	Hs_PURINOCEPTORY14_1_SG
$P2X_1$	Hs_PURINOCEPTORX1_1_SG
$P2X_2$	Hs_PURINOCEPTORX2_1_SG
$P2X_3$	Hs_PURINOCEPTORX3_1_SG
$P2X_4$	Hs_PURINOCEPTORX4_1_SG
$P2X_5$	Hs_PURINOCEPTORX5_1_SG
$P2X_6$	Hs_PURINOCEPTORX6_1_SG
$P2X_7$	Hs_PURINOCEPTORX7_1_SG

All primers were from Qiagen.

Table S3. Ca^{2+} signals evoked by P2Y-selective agonists in cultured human foetal astrocytes

Agonist	Activity[1]	$\Delta[Ca^{2+}]_i$		
		pEC_{50}, M	Maximal response, nM	n
ATP	1, 2, 6, 11	5.94 ± 0.03	180 ± 14	8
ADP	1, 6	6.00 ± 0.11	127 ± 10	3
MRS2365	1	6.20 ± 0.19	97 ± 8	5
UDP	6	ND	1 ± 1	3
UTP	2, 11	4.86 ± 0.18	58 ± 4	5
2'-thio-UTP	2	4.87 ± 0.47	71 ± 3	3
2’-amino-UTP	2	4.57 ± 0.22	51 ± 13	3
NF546	11	ND	4 ± 3	3

[1]Activity at the four P2Y subtypes for which mRNA was detected in human astrocytes ($P2Y_1$, $P2Y_2$, $P2Y_6$ and $P2Y_{11}$) (Fig. 1E). ND, not determined.

Supporting references

Syed, N.-i.-H. and Kennedy, C. (2012) Pharmacology of P2X receptors. *WIREs Membr Transp Signal,* **1**, 16-30.

von Kugelgen, I. and Hoffmann, K. (2015) Pharmacology and structure of P2Y receptors. *Neuropharmacol.,* **104**, 50-61.

A

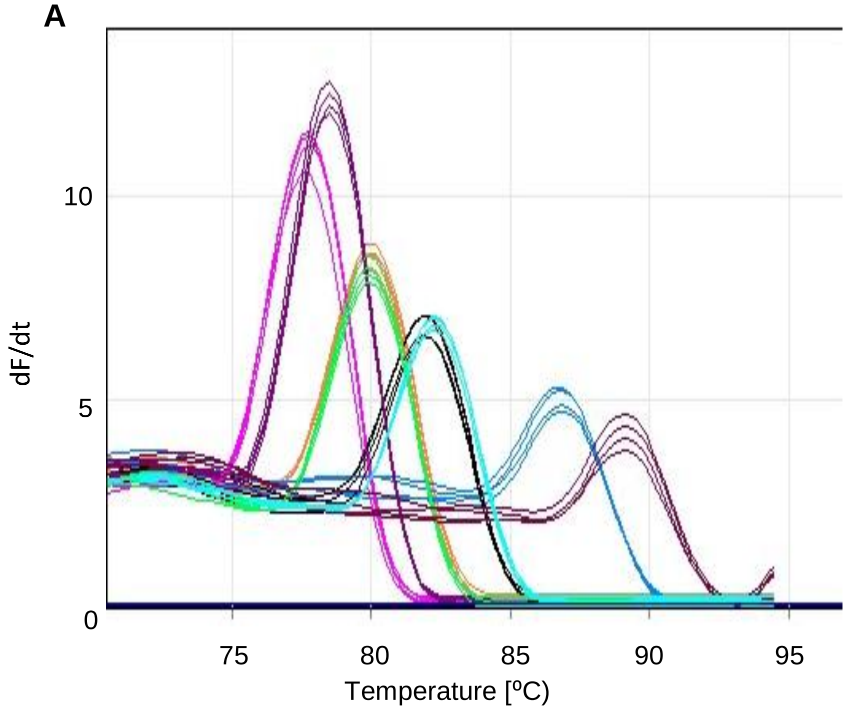

Colour	Gene	Replicate Peak
	$P2Y_6$	80.0
	$P2Y_{11}$	86.8
	$P2Y_{12}$	77.7
	$P2Y_{13}$	82.0
	$P2Y_{14}$	78.5
	$P2Y_2$	80.0
	$P2Y_4$	89.0
	$P2Y_1$	82.3

B

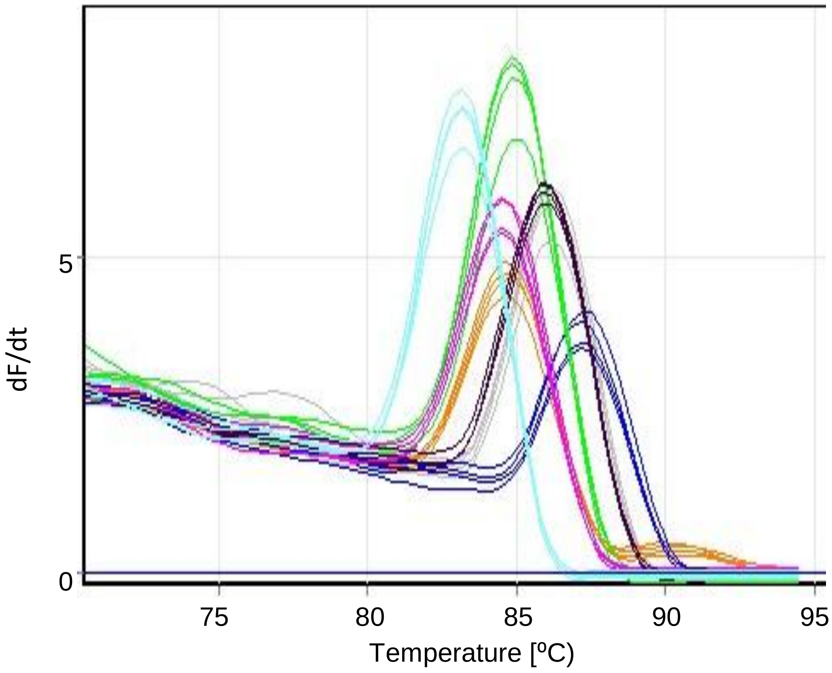

Colour	Gene	Replicate Peak
	$P2X_1$	86.2
	$P2X_2$	84.7
	$P2X_3$	87.2
	$P2X_4$	84.8
	$P2X_6$	84.5
	$P2X_5$	86.0
	$P2X_7$	83.2

Fig. S1 Melting curves for qPCR analyses of the expression of purinoreceptor subtypes. (A, B) Traces show the rate of change of SYBR green fluorescence (dF/dt) as a function of temperature for the primers used to selectively amplify mRNA (Table S2) from BioBank pooled cDNA for the indicated P2Y receptors (A) or P2X receptors (B). Results show four separate determinations for each primer pair. The mean peak of each melting curve is shown in the tables.

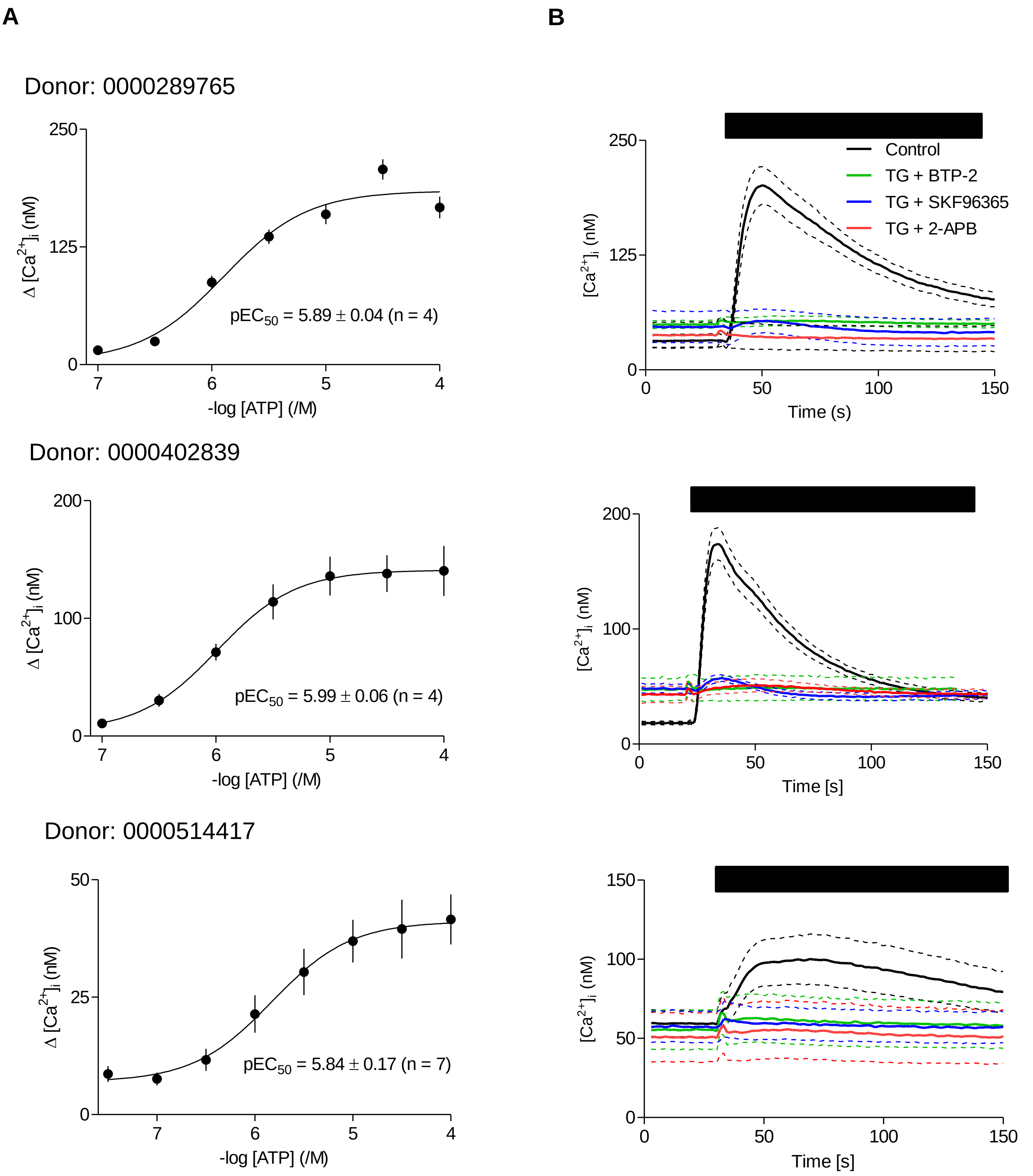

Fig. S2 ATP evokes Ca^{2+} signals through P2Y receptors in astrocytes from three donors. (A) Concentration-dependent effects of ATP on the peak increase in $[Ca^{2+}]_i$ in cells from the indicated donors. pEC_{50} values are shown for each donor. Results show means ± SEM, n = 4-7. Pooled results from the first 2 donors are shown in Fig. 1B. (B) Traces show responses to ATP (100 μM, solid bar) added to cells in HBS alone or after treatment with thapsigargin (5 μM, 15 min) with the indicated inhibitors of SOCE (concentrations defined in Fig. 2D). Results show mean (solid line) ± SEM (or range for n = 2, dashed lines), n = 2-7. Summary results from the first 2 donors are shown in Fig. 2F.

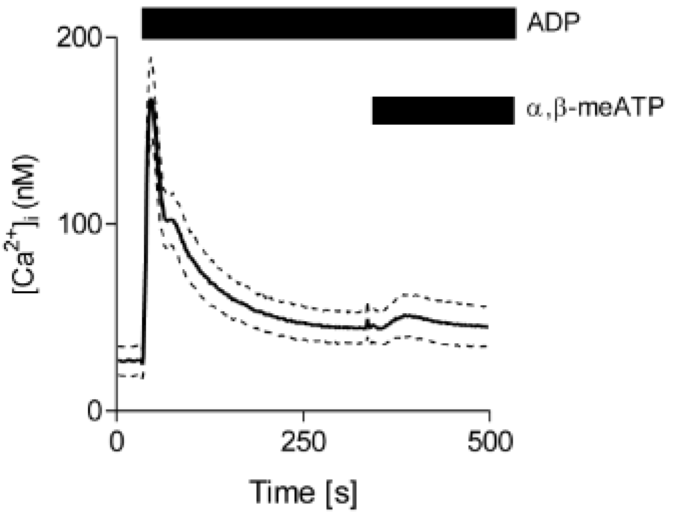

Fig. S3 Stimulation of P2Y receptors does not cause translocation of functional P2X receptors to the plasma membrane. Populations of fluo-8-loaded astrocytes in HBS were stimulated with ADP (100 µM) to selectively activate P2Y receptors and then with α,β-meATP (30 µM) to allow selective activation of P2X receptors. Traces show $[Ca^{2+}]_i$ as means (solid line) ± SEM (dashed line), n = 3.

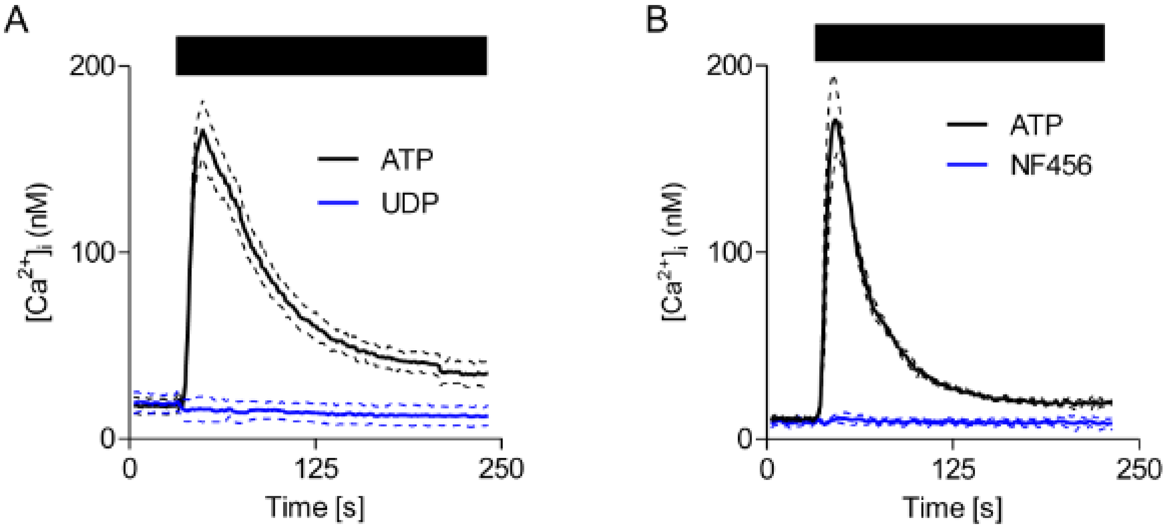

Fig. S4 Neither $P2Y_6$ nor $P2Y_{11}$ receptors evoke Ca^{2+} signals in cultured human foetal astrocytes. (A,B) Responses of cell populations in HBS to ATP (100 µM) or UDP (300 µM, agonist of $P2Y_6$ receptor) (A), or NF456 (3 µM, agonist of $P2Y_{11}$ receptor) (B), each applied for the periods shown by solid bars. Traces show $[Ca^{2+}]_i$ as means (solid line) ± SEM (dashed line), n = 3.

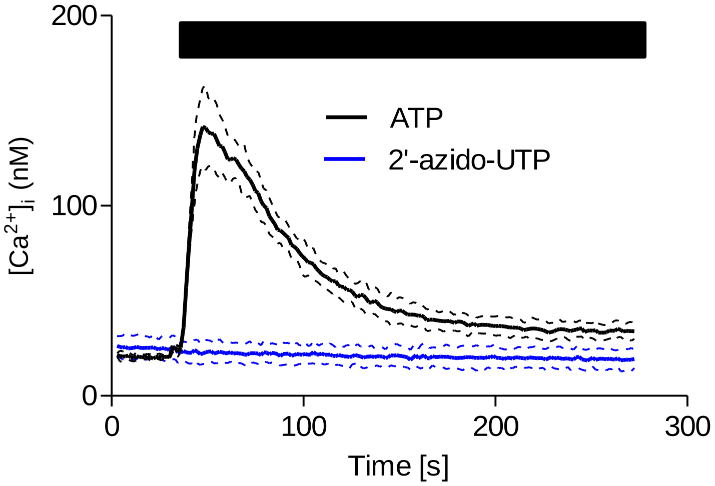

Fig. S5 2´-azido-UTP does not evoke Ca^{2+} signals. Responses of cell populations in HBS to ATP (100 µM) or 2´-azido-UTP (100 µM, a selective agonist of $P2Y_4$ receptors). Traces show $[Ca^{2+}]_i$ as means (solid lines) ± SEM (dashed lines), n = 4.

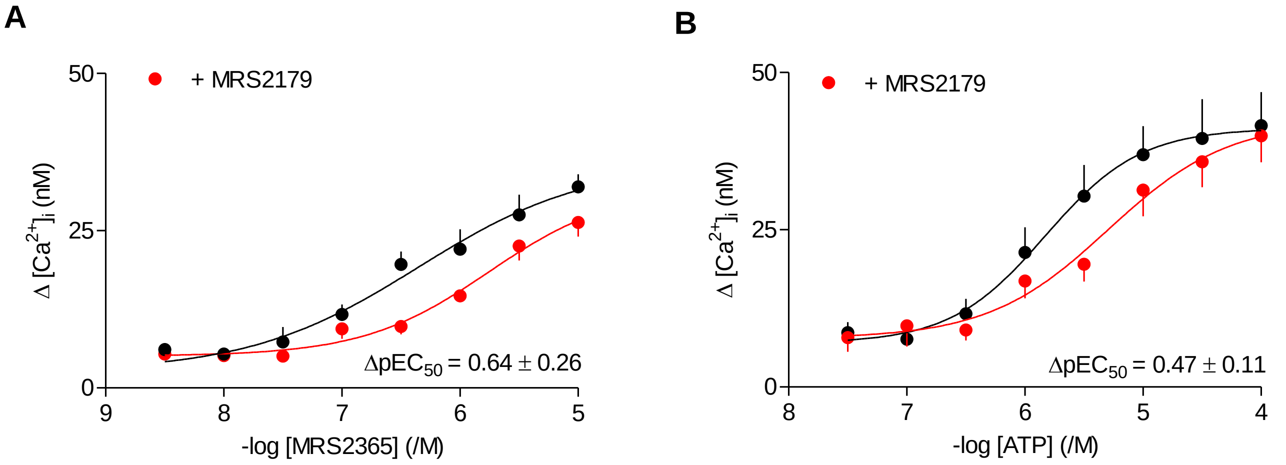

Fig. S6 Effects of MRS2179, a selective antagonist of $P2Y_1$ receptors, on the Ca^{2+} signals evoked by ATP and MRS2365. (A, B) Summary results (mean ± SEM, n = 6-7) from astrocytes taken from donor 0000514417 show the effects of MRS2179 (5 µM, added 5 min before stimulation) on the peak Ca^{2+} signals evoked by MRS2365 (A) or ATP (B). ΔpEC_{50} values are shown, where $\Delta pEC_{50} = p_{EC_{50}}^{+MRS2179} - p_{EC_{50}}^{control}$. The results show that the selective $P2Y_1$ agonist, MRS2365, evokes smaller Ca^{2+} signals that are more susceptible to inhibition by MRS2179 than those evoked by ATP. Traces show $[Ca^{2+}]_i$ as means (solid lines) ± SEM (dashed lines).
